# Circulating miR‐130 and its target PPAR‐γ may be potential biomarkers in patients of coronary artery disease with type 2 diabetes mellitus

**DOI:** 10.1002/mgg3.909

**Published:** 2019-08-01

**Authors:** Yonggang Yuan, Wanzhong Peng, Yongxing Liu, Zesheng Xu

**Affiliations:** ^1^ Department of Cardiology Cangzhou Central Hospital Cangzhou Hebei China

**Keywords:** biomarker, coronary artery disease, miR‐130, PPAR‐γ, type 2 diabetes mellitus

## Abstract

**Background:**

Patients of coronary artery disease (CAD) with type 2 diabetes mellitus (DM2) show increased mortality risk than CAD patients without DM2, while few biomarkers can be used to discriminate them.

**Methods:**

Fifty‐nine patients of CAD with DM2 (DM2‐CAD group), 79 patients of CAD without DM2 (CAD group), and 63 healthy control subjects were recruited. Circulating miR‐130 (miR‐130a and miR‐130b) and PPAR‐γ (peroxisome proliferator‐activated receptor gamma) were measured and their Pearson correlation was analyzed. 3′ UTR binding prediction and luciferase assay were used to determine the target relationship between miR‐130 and PPAR‐γ. Receiver operating characteristics (ROC) analysis was performed to test the discrimination ability of miR‐130 between DM2‐CAD and CAD groups.

**Results:**

miR‐130a and miR‐130b showed decreased expression in DM2‐CAD group when compared with the CAD group and health control. Both bioinformatics and luciferase assays showed that miR‐130 could bind the 3′ UTR of *PPAR‐γ*. Furthermore, miR‐130 negatively correlated with PPAR‐γ in both CAD and DM2‐CAD group in Pearson's coefficient analysis. Both miR‐130a and miR‐130b were able to discriminate DM2‐CAD group from CAD group and control subjects.

**Conclusion:**

Circulating miR‐130 may regulate the expression of PPAR‐γ and can be used as a biomarker to discriminate DM2‐CAD from CAD.

## 1 INTRODUCTION

Coronary artery disease (CAD), which includes angina, myocardial infarction, and sudden cardiac death, is the predominant cause of morbidity and mortality (Haffner, Lehto, Rönnemaa, Pyörälä, & Laakso, 1998; Wong, 2014). Diabetes mellitus (DM), especial type 2 DM, which is closely associated with clustered risk factors, such as hypertension, hypercholesterolemia, obesity, and smoking, can show a two‐ to fourfolds mortality risk for CAD (Aronson & Edelman, 2014; Beller et al., 2018). Although there has been considerable progress in the diagnosis and treatment for CAD and its complications, coronary event rates remain high among patients with DM2 (Preis et al., 2009). It is a clinical urgency to develop noninvasive diagnostic biomarker and new treatment strategy to decrease CAD morbidity and mortality. Specific microRNAs (miRNAs) expression signatures have been linked to the pathology of CAD, for miRNAs could function as posttranscriptional modulators associated with physiological and pathological processes linked to CAD (Izawa & Amano, 2015; Malik et al., 2017). It is worth noting that in addition to their intracellular function, extracellular exported or released miRNAs can circulate in a remarkably stable form within the blood and mediate the intercellular communication (Guo & Huang, 2018), which attracts more attention as novel noninvasive biomarkers for the early diagnosis and differential diagnosis of CAD and its complications such as DM2 (Borghini, 2018; Jia et al., 2017). In this study, circulating miR‐130 and its target PPAR‐γ (peroxisome proliferator‐activated receptor gamma) are investigated to distinguish DM2‐CAD (CAD with DM2) group from CAD (CAD without DM2) group. Our results suggest that circulating miR‐130 may regulate the expression of PPAR‐γ and can be used as sensitive noninvasive biomarkers of CAD differential diagnose and monitoring.

## 2 MATERIALS AND METHODS

### 2.1 Ethical compliance

This study was approved by the Ethics Committee of Cangzhou Central Hospital, and written informed consents were provided by all the participants.

### 2.2 Participants recruited

All the participants were recruited from the cardiology department of Cangzhou Central Hospital between January 2015 and January 2017 who were further classified into three groups: healthy control group, DM2‐CAD group, and CAD group. CAD was confirmed with quantitative coronary angiography based on a modified AHA/ACC classification as at least one primary epicardial vessel with ≥50% stenosis (Kim et al., 2010). The associated complication of DM2 was self‐reported and diagnosed in accordance with the World Health Organization (WHO) criteria (Zhao, Zhu, Song, & Li, 2015): fasting blood glucose (FG) levels ≥7.0 mmol/L, or a 2‐hour oral glucose tolerance test (OGTT) ≥11.1 mmol/L in the presence of symptoms and glycated hemoglobin (HbA1c) levels >6.5%. Patients with the history of severe hepatic dysfunction, systemic disorder or inflammatory disease, and malignant diseases were excluded.

### 2.3 Reverse transcription polymerase chain reaction (RT‐PCR)

About 5–10 ml venous blood was extracted and total RNA was isolated using PAXgene Blood miRNA Kit (Qiagen) according to the manufacturer's recommendation. miRNAs were isolated in the serum with the mirVanaTM miRNA Isolation Kit (Ambion). Designed mature miR‐130a‐ and miR‐130b‐ specific stem loop‐RT primers (referred to TaqMan assays of 000454 for hsa‐miR‐130a and 000456 for hsa‐miR‐130b), endogenous control miR U6 primers (referred to TaqMan assays of 001006 for U6), PPAR‐γ primers (forward primer, TCGCTGATGCACTGCCTATG; reverse primer, GAGAGGTCCACAGAGCTGATT), and β‐actin primers (forward primer, AAATCTGGCACCACACCTTC; reverse primer, GGGGTGTTGAAGGTCTCAAA) were synthesized. Then, the NCode™ miRNA First‐Strand cDNA Synthesis Kit (Invitrogen) and High‐Capacity cDNA Reverse Transcription kits (Applied Biosystems) were used to get reverse‐transcribed cDNA. The real‐time PCR reaction incubated in a 96‐well plate was set up in a 20‐µl volume using SYBR Green Master Mix (TaKaRa Biotechnology Co., Ltd). The PCR reaction was performed using an ABI PRISM 7500 system (Applied Biosystems) with an initial denaturation of 95°C for 10 min, followed by 40 cycles of 95°C for 15 s and 60°C for 1 min. 2^−∆∆ CT^ method was adopted to quantify the relative expression.

### 2.4 Enzyme‐linked immunosorbent assay (ELISA)

The concentration of PPAR‐γ in the serum was detected with ELISA kits (eBioscience) according to the manufacturer's instructions. All samples and standards were assayed with a microplate reader (SpectraMax M5, Molecular Devices) at a wavelength of 450 nm.

### 2.5 3′ UTR luciferase reporter assay

The DNA segments containing wild‐type and mutated 3′ UTR sequences of PPAR‐γ were synthesized and inserted into the pGL3‐CMV vector (Catalog # E1751; Promega) at the downstream of firefly luciferase open reading frame, using BamHI and SalI restriction sites. The pGL3‐basic construct and hsa‐miR‐130a‐3p mimic (Catalog # MC10506; Thermo Fisher) or scrambled control were transfected into HEK293T cells with Lipofectamine 2000 (Invitrogen). Luciferase activities were measured by a Dual‐Luciferase Reporter Assay System (Catalog # E1910; Promega) 48 hr later after transfection, and firefly luciferase activity was normalized to Renilla luciferase activity.

### 2.6 Receiver operating characteristic (ROC) analysis

Receiver operating characteristic (ROC) curve [i.e., sensitivity vs. (1 − specificity)] was utilized to calculate the posterior probabilities previously derived and to represent the diagnostic performance for miR‐30 to discriminate CAD group from DM2‐CAD group. And, the area under the ROC curve (AUC) presented a global measure of the clinical efficiency over a range of test cutoff points on the ROC curve. The AUC of the ROC analysis of 0.83–0.99 indicated the highly accurate performance of the test.

### 2.7 Statistical analysis

Results were shown as mean ± standard deviation (*SD*). *p* < .05 was considered to be significant.

## 3 RESULTS

### 3.1 Clinical characteristics of the participants

Sixty‐three healthy control subjects, 79 CAD subjects, and 59 DM2‐CAD subjects were enrolled and the relevant clinical data were shown in Table 1. Gender and age distribution showed no statistical difference (*p* > .05) in all the groups, whereas blood glucose level (FG), diabetes duration, hemoglobin A1c (HbA1c), low‐density lipoprotein cholesterol (LDL‐C), and total cholesterol (TC) differed significantly in CAD group and DM2‐CAD group when compared with healthy control. High‐density lipoprotein cholesterol (HDL‐C) and triglyceride (TG) only differed significantly in DM2‐CAD group when compared with healthy control. In addition, serum creatinine (Cr.) and estimated glomerular filtration rate (eGFR), which can indicate renal function, showed no significant differences in CAD group and DM2‐CAD group when compared with healthy control.

**Table 1:** Clinical characteristics of each group

	Control	CAD	DM2‐CAD
Gender (M/F)	63 (30/33)	79 (42/37)	59 (28/31)
Age (years)	56 ± 9.6	61 ± 10.8	59 ± 11.7
FG (mmol/L)	4.95 ± 0.85	9.12 ± 2.21**	11.67 ± 2.72**
HbA1c (%)	3.18 ± 1.3	7.55 ± 7.9**	10.1 ± 6.9**
Hemoglobin (g/dl)	12.87 ± 1.6	13.71 ± 1.9	12.45 ± 2.1
WBC (×10^9^/L)	8.1 ± 4.1	7.7 ± 2.7	7.9 ± 3.9
Platelet count (×10^9^)	269 ± 50.6	251 ± 64.5	259 ± 62.8
MPV (fL)	9.97 ± 1.06	10.9 ± 1.14*	11.2 ± 1.26*
Diabetes duration (years)	‐	14.5 ± 5.5**	20.8 ± 8.3**
BMI (kg/m^2^)	23.2 ± 4.1	26.4 ± 3.8*	27.2 ± 5.1*
Smoking (%)	15 (23.80)	17 (21.51)	16 (27.11)
Hypertension (%)	23 (36.50)	24 (30.37)	21 (35.59)
Hyperlipidemia (%)	20 (31.74)	25 (31.64)	22 (37.29)
Mean BP (mmHg)	85.4 ± 2.7	89.5 ± 9.4*	95.2 ± 7.7*
LDL‐C (mmol/L)	2.38 ± 0.7	2.98 ± 1.3*	5.35 ± 1.5**
HDL‐C (mmol/L)	1.38 ± 0.4	1.10 ± 0.29	0.88 ± 0.38*
TC (mmol/L)	4.08 ± 0.9	4.81 ± 1.8*	7.15 ± 2.4**
TG (mmol/L)	1.48 ± 0.6	1.55 ± 0.8	2.48 ± 1.08**
ACR (mg/mmol)	0.67 ± 0.28	0.88 ± 0.34	0.94 ± 0.57*
Serum creatinine (μm/L)	54.7 ± 10.8	59.1 ± 13.9	64.8 ± 15.3
eGFR (ml/min/1.73m^2^)	103 ± 13.4	102 ± 11.5	104 ± 12.2

Note

Data are presented as number (percentage) for categorical data or mean ± standard deviation (*SD*) for parametrically distributed data.

Abbreviations: ACR: albumin/creatinine ratio; BMI, body mass index; BP, blood pressure; CAD, coronary artery disease; DM2, type 2 diabetes mellitus; eGFR, estimated glomerular filtration rate; FG, fasting glucose; HbA1c, glycated hemoglobin; HDL‐C, high‐density lipoprotein cholesterol; LDL‐C, low‐density lipoprotein cholesterol; MPV, mean platelet volume; TC, total cholesterol; TG, triglyceride; WBC, white blood cell.

* *p* < .05,

** *p* < .01 compared to control group.

### 3.2 Opposite expression pattern of circulating miR‐30 and PPAR‐γ in coronary artery disease

**Figure 1:**
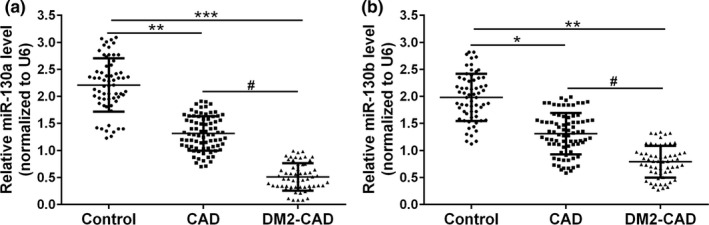
The levels of circulating miR‐130 in patients’ serum. Secretion of miR‐130a (a) and miR‐130b (b) levels was detected with qRT‐PCR analysis in control, CAD group, and DM2‐CAD group. The expressions were normalized to U6 RNA and analyzed with the 2^–ΔΔCt^ method. *p < .05, **p < .01, and ***p < .001 compared to control group, #*p* < .05 compared to CAD group

**Figure 2:**
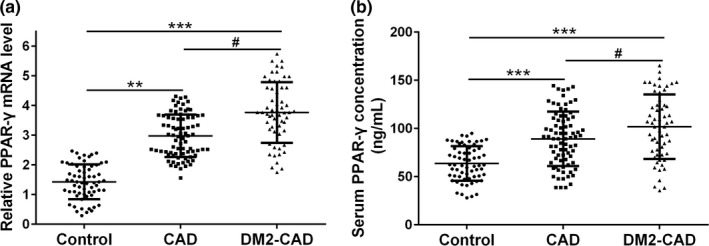
The levels of PPAR‐γ in patients’ serum. (a) qRT‐PCR was used to measure the mRNA expression of PPAR‐γ in different groups. The data were normalized to β‐actin. (b) Serum PPAR‐γ concentration level was analyzed by ELISA in different groups. Data were shown as mean ± *SD*, **p < .01, and ***p < .001 compared to control group, #*p* < .05 compared to CAD group

Circulating miR‐130 was significantly downregulated in CAD group compared with healthy control subjects (miR‐130a, 2.3‐fold, *p* < .01; miR‐130b, 1.5‐fold, *p* < .05), while such decrease can also be observed in DM2‐CAD group compared with the CAD group (miR‐130a, 2.4‐fold, *p* < .05; miR‐130b, 1.6‐fold, *p* < .05) (Figure 1a and b). All of these data suggested that the secretion of miR‐130 was negatively correlated with the risk of coronary events. Consistent with anticipation, PPAR‐γ mRNA levels were upregulated by 2.0‐fold in CAD group (*p* < .01) and 2.5‐fold in DM2‐CAD group (*p* < .001) compared with healthy subjects (Figure 2a). In accordance with the mRNA expression, the secretion of PPAR‐γ also showed a similar pattern (Figure 2b).

### 3.3 miR‐130 can directly target PPAR‐γ

**Figure 3:**
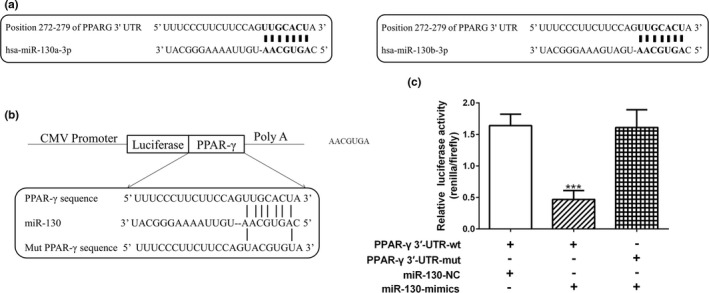
PPAR‐γ is a potential miR‐130 target. (a) The 3′‐UTR sequence of PPAR‐γ was screened and aligned to identify complementarity between PPAR‐γ and both miR‐130a and miR‐130b. (b) The suspected binding of miR‐130 with the wild‐type 3′‐UTR region of PPAR‐γ mRNA is shown. A mutated 3′‐UTR of PPAR‐γ is also shown. (c) Dual‐luciferase reporter gene assay showed the direct functional interactions between PPAR‐γ and miR‐130. Data were shown as mean ± *SD*, firefly luciferase activity was normalized to Renilla luciferase activity, ***p < .001 compared to wild‐type 3′‐UTR of PPAR‐γ cotransfected with control miRNA

Online 3′‐UTR binding site prediction databases (Microcosm and Targetscan) were used to perform overlap screen analysis. A conserved binding site within PPAR‐γ was found which indicated that miR‐130 could directly target PPAR‐γ (Figure 3a). Then, nine complementary nucleotides in the 3′‐UTR regions of PPAR‐γ were mutated and fused into the luciferase coding region, which was further cotransfected with miR‐130 mimic into HEK293T cells (Figure 3b). When miR‐130 mimic and wild‐type PPAR‐γ 3′‐UTR were cotransfected, the relative luciferase activity was significantly decreased compared with control miRNA. While such an effect was not observed after miR‐130 mimic and mutant PPAR‐γ 3′‐UTR cotransfection. All of these data indicated that miR‐130 could directly target and inhibit the expression of PPAR‐γ (Figure 3c).

### 3.4 miR‐130 negatively correlates with PPAR‐γ

The association of circulating miR‐130 with PPAR‐γ in the control group, CAD group, and DM2‐CAD group was performed with Pearson's coefficient analysis. As shown in Table 2, both miR‐130a and miR‐130b were negatively correlated with PPAR‐γ in both DM2‐CAD group and CAD group. Neither miR‐130a nor miR‐130b showed significant correlation with PPAR‐γ in the control subjects.

**Table 2:** Pearson's coefficient correlation analysis of miR‐130a and miR‐130b with PPAR‐γ

r		miR‐130a	miR‐130b
Control	PPAR‐γ mRNA level	0.189	0.231
	Serum PPAR‐γ concentration	−0.148	0.173
CAD	PPAR‐γ mRNA level	−0.521**	−0.446*
	Serum PPAR‐γ concentration	−0.484**	−0.595**
DM2‐CAD	PPAR‐γ mRNA level	−0.772***	−0.682***
	Serum PPAR‐γ concentration	−0.573**	−0.397*

* *p* < .05,

** *p* < .01 and

*** *p* < .001.

### 3.5 Diagnostic value of miR‐130 in CAD patients

**Figure 4:**
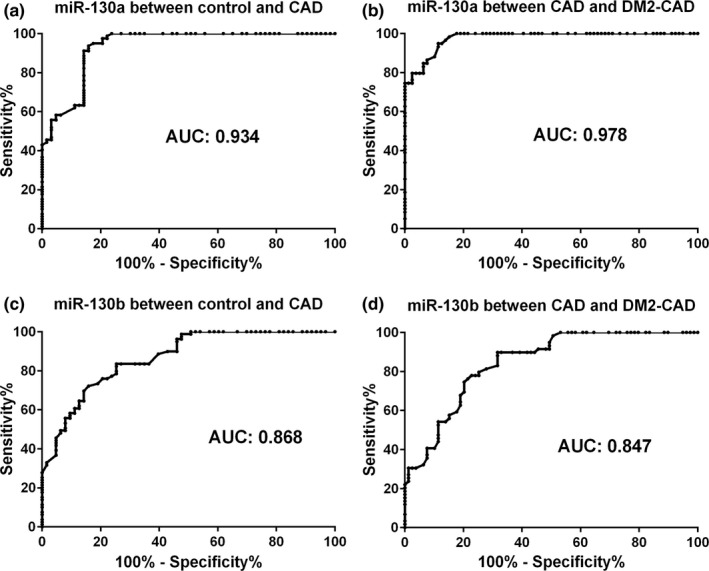
The biomarker potential of circulating miR‐130. Receiver operating characteristics (ROC) analysis was applied to evaluate the ability of circulating miR‐130a and miR‐130b to discriminate different groups. (a) miR‐130a distinguished CAD patients from controls with area under curve (AUC) of 0.934 (Confidence interval [IC]: 0.892–0.975; *p* < .001). (b) miR‐130a distinguished CAD patients from DM2‐CAD with AUC of 0.978 (Confidence interval [IC]: 0.960–0.996; *p* < .001). (c) miR‐130b distinguished CAD patients from controls with AUC of 0.868 (Confidence interval [IC]: 0.810–0.925; *p* < .001). (d) miR‐130b distinguished CAD patients from DM2‐CAD with AUC of 0.847 (Confidence interval [IC]: 0.784–0.909; *p* < .001)

ROC analysis showed that miR‐130a could distinguish CAD group from the control group and DM2‐CAD group from CAD group, with an AUC of 0.934 and 0.978, respectively (Figure 4a and b). Similarly, miR‐130b also distinguished CAD patients from controls and DM2‐CAD patients from CAD, with an AUC of 0.868 and 0.847 (Figure 4c and d). Such data indicated that miR‐130 could be used as a biomarker to distinguish CAD from DM2‐CAD and to predict the risk of coronary events.

## 4 DISCUSSION

A significant decrease in the circulating miR‐130 in CAD patients with DM2 compared with CAD patients without DM2 and health control can be observed, which indicates a negative correlation with the risk of coronary events. Pearson's correlation analysis shows a significant and negative correlation between miR‐130 and PPAR‐γ in both CAD and DM2‐CAD groups. Both bioinformatics and luciferase assays testify that miR‐130 could directly target the 3′ UTR of PPAR‐γ and mediate the relevant transcription. The discrimination between CAD patients and control subjects or CAD patients with DM2 and CAD patients without DM2 can be assayed by miR‐130 in ROC analysis. CAD can be considered as serious coronary events due to plaque formation and subsequent coronary arteries obstruction (Hansson, 2005; Pepine & King, 2018). During the development of plaque, miRNAs can be released into the circulation from the cellular components of the plaque which may be used as biomarkers to predict CAD (Izawa & Amano, 2015; Jansen et al., 2017). Several other studies also demonstrate that miR‐130 might be independently connected with the presence and severity of CAD (Chu & Zhou, 2015; Jansen et al., 2014). While no mechanistic analyses were reported in these studies. It is worth noting that circulating miR‐130 level is downregulated in CAD patients with DM2, which is consistent with the previous observation that serum miR‐130b level is significantly decreased in DM2 patients compared with control and correlated with the severity of diabetic nephropathy (Lv et al., 2015). It is further testified that miR‐130b could promote obesity‐associated adipose tissue inflammation and insulin resistance in diabetes mice through alleviating M2 macrophage polarization via repression of PPAR‐γ (Zhang & Zhou, 2016). Whether such a mechanism also happened in humans needs further investigation. It is worth noting that miR‐130 is upregulated in DM2 patients when compared with normal glucose tolerance (NGT) or impaired glucose tolerance (IGT) persons in Indian population (Prabu et al., 2015), such discrepancy indicates that multicenter investigations are needed to decipher the relevant mechanism involved.

PPAR‐γ has mostly been considered as a crucial metabolic sensor connected with glucose and lipid metabolism homeostasis, lipid storage, and adipogenesis (Lehrke & Lazar, 2005; Lim, 2018). PPAR‐γ may attenuate plaque stabilization by reducing matrix metallopeptidase 9 (MMP‐9) expression and promote the formation of foam cell by stimulating the intake of oxidized LDL (Kersten, Desvergne, Desvergne, & Wahli, 2000) which may also be offset by PPAR‐γ–dependent or independent counter‐regulatory mechanisms (Chrisman et al., 2018; Wojtkowska et al., 2014). All of these suggest that PPAR‐γ can link altered lipid and glucose metabolism with CAD development especially in DM. Moreover, human genetic studies recently reveal that functional changes in this nuclear receptor are strongly associated with CAD (Amoruso & Bardelli CFresu, 2009; Li, Zhu, & Ding, 2015). A polymorphism of C161T substitution in exon 6 of the PPAR‐γ gene may affect the secretion of pro‐inflammatory cytokines and affect CAD susceptibility (Qian et al., 2016).

Our data indicate that miR‐130 could directly bind to the PPAR‐γ mRNA 3’ UTR region to take part in the progress of CAD and the expression of miR‐130 and PPAR‐γ shows a negative correlation in CAD patients with DM2 and CAD patients without DM2. Circulating miR‐130 can act as not only a potential prognostic parameter but also a biomarker to distinguish CAD patients with DM2 from CAD patients without DM2. However, a relatively small size sample was enrolled in the present study and the utility of miR‐130 to discriminate will need further confirmation.

## 5 CONCLUSION

miR‐130 shows a negative correlation with the risk of coronary events and can be used as a biomarker to distinguish CAD patients with DM2 from CAD patients without DM2.

## CONFLICT OF INTEREST

The authors declare that there is no conflict of interests.
